# Diabetic Foot Ulcer Identification: A Review

**DOI:** 10.3390/diagnostics13121998

**Published:** 2023-06-07

**Authors:** Sujit Kumar Das, Pinki Roy, Prabhishek Singh, Manoj Diwakar, Vijendra Singh, Ankur Maurya, Sandeep Kumar, Seifedine Kadry, Jungeun Kim

**Affiliations:** 1 Department of Computer Science and Engineering, ITER, Siksha ‘O’ Anusandhan University, Bhubaneswar 751030, India; 2 Department of Computer Science and Engineering, National Institute of Technology, Silchar 788010, India; 3 School of Computer Science Engineering and Technology, Bennett University, Greater Noida 201310, India; 4 Computer Science and Engineering Department, Graphic Era Deemed to Be University, Dehradun 248002, India; 5 School of Computer Science, University of Petroleum and Energy Studies, Dehradun 248007, India; 6 Department of Computer Science & Engineering, Maharaja Surajmal Institute of Technology, Delhi 110058, India; 7 Department of Applied Data Science, Noroff University College, 4612 Kristiansand, Norway; 8 Artificial Intelligence Research Center (AIRC), Ajman University, Ajman 346, United Arab Emirates; 9 Department of Electrical and Computer Engineering, Lebanese American University, Byblos P.O. Box 13-5053, Lebanon; 10 MEU Research Unit, Middle East University, Amman 11831, Jordan; 11 Department of Software and CMPSI, Kongju National University, Cheonan 31080, Republic of Korea

**Keywords:** diabetic foot ulcer, convolutional neural network, deep learning, identification

## Abstract

Diabetes is a chronic condition caused by an uncontrolled blood sugar levels in the human body. Its early diagnosis may prevent severe complications such as diabetic foot ulcers (DFUs). A DFU is a critical condition that can lead to the amputation of a diabetic patient’s lower limb. The diagnosis of DFU is very complicated for the medical professional as it often goes through several costly and time-consuming clinical procedures. In the age of data deluge, the application of deep learning, machine learning, and computer vision techniques have provided various solutions for assisting clinicians in making more reliable and faster diagnostic decisions. Therefore, the automatic identification of DFU has recently received more attention from the research community. The wound characteristics and visual perceptions with respect to computer vision and deep learning, especially convolutional neural network (CNN) approaches, have provided potential solutions for DFU diagnosis. These approaches have the potential to be quite helpful in current medical practices. Therefore, a detailed comprehensive study of such existing approaches was required. The article aimed to provide researchers with a detailed current status of automatic DFU identification tasks. Multiple observations have been made from existing works, such as the use of traditional ML and advanced DL techniques being necessary to help clinicians make faster and more reliable diagnostic decisions. In traditional ML approaches, image features provide signification information about DFU wounds and help with accurate identification. However, advanced DL approaches have proven to be more promising than ML approaches. The CNN-based solutions proposed by various authors have dominated the problem domain. An interested researcher will successfully be able identify the overall idea in the DFU identification task, and this article will help them finalize the future research goal.

## 1. Introduction

Diabetes mellitus, often known as diabetes, is a metabolic disorder characterized by elevated blood glucose levels (hyperglycemia) [1]. Pancreatic β-cells secrete insulin, which transfers sugar from the blood into the body’s cells, where it is stored or utilized for energy. However, in an individual with diabetes, the pancreas either cannot produce sufficient insulin or the body loses its ability to use the insulin produced [2]. Based on the degree of hyperglycemia, diabetes can be classified as:

- Type 1 diabetes, which affects 10% of diabetic population, causes the loss of cells, producing insulin in the pancreas.
- Type 2 diabetes causes blood sugar levels to rise as the body becomes insulin-resistant.
- Gestational diabetes is occurs during pregnancy due the insulin-blocking hormones produced by the placenta.

**Figure 1:**
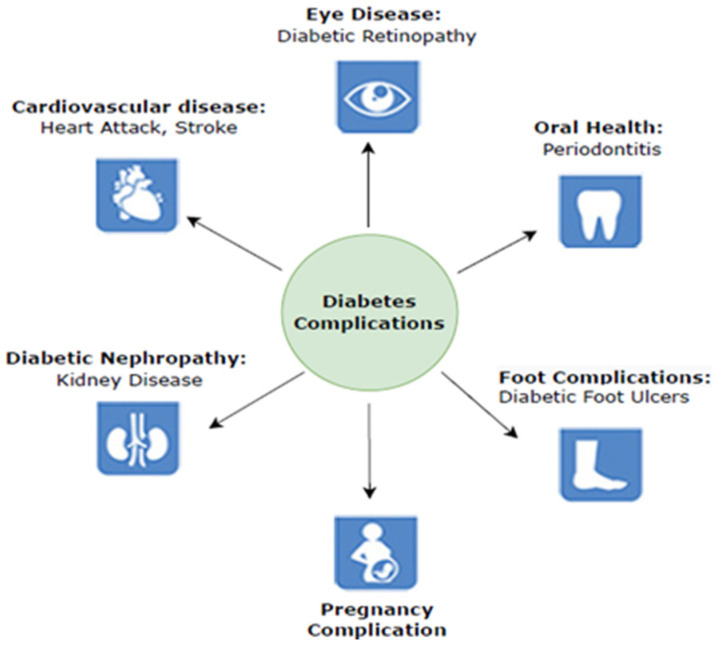
Diabetes complications [3].

In a 2021 study, the International Diabetes Federation stated that diabetes affects 537 million persons between the ages of 20 and 79. This number is estimated to rise to approximately 783 million by 2045 [3]. However, one in two diabetes cases is undiagnosed, resulting in 6.7 million deaths. Diabetes is highly likely to cause various life-threatening conditions in the undiagnosed population [4]. Cardiac diseases, heart attacks, kidney diseases, retinopathy, neurological complications, foot injuries hearing loss, vision problems, bacterial and tinea infections, depression, insomnia, and dementia are examples of consequences. According to the IDF, the major complications related to diabetes are shown in Figure 1. This work was focused on diabetic foot complications caused by damages to blood vessels and peripheral nerves.

**Figure 2:**
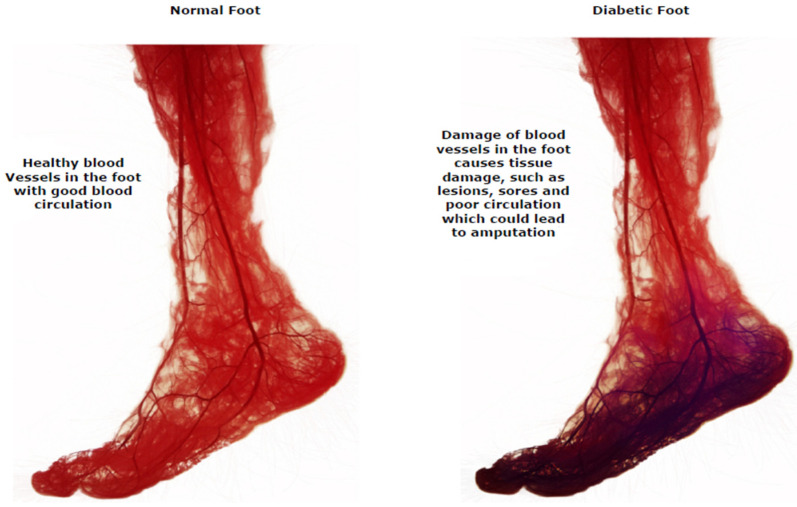
Blood circulation in a healthy foot and a DFU foot [11].

Diabetic foot ulcers (DFUs) are a common consequence of poorly treated diabetes that can harm the feet, right down to the bones. Early indications of diabetic foot ulcers include unusual swelling, exudate maceration, redness, itching, irritation, and odor [5]. A black tissue, called eschar, surrounding the ulcer area is the most visible sign of serious DFU [6]. People with type 2 diabetes often deal with this complication, fighting off infections due to DFU. Diabetic foot problems are a prominent cause of non-traumatic lower extremity amputations globally. DFUs account for nearly 85% of lower limb amputations [7,8,9]. Diabetic peripheral neuropathy, structural abnormalities of foot deformities, and peripheral artery occlusive disease are the most frequent risk factors for DFU. Foot ulcers are the most common reason diabetic people are admitted to hospitals. The comprehensive evaluation and risk assessment of a diabetic individual’s feet necessitate a multidisciplinary foot care team [10,11,12,13,14]. Treatment must involve a patient’s medical records, lab test results, and assessments of their subcutaneous, nervous system, cardiovascular, musculoskeletal, rheumatological, and vascular states. In current clinical practice, the severity of DFUs are inspected visually by podiatrists and healthcare professionals using manual measurement tools, with additional examinations conducted using technology such as X-rays, computed tomography scans, magnetic resonance imaging, and ultrasound tests [15,16,17,18]. The difference between a normal foot and one with a DFU in terms of blood circulation is shown in Figure 2.

**Figure 3:**
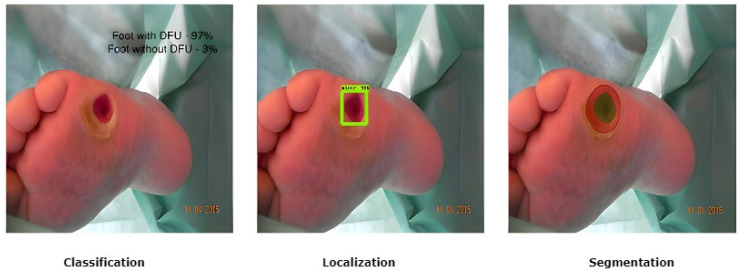
Different DFU tasks performed by medical imaging and computer vision [25].

The treatment of DFU is costly [19]. The cost of diagnosis, regular and periodic check-ups, the continuous use of expensive medications, and proper healthcare, including the maintenance of personal hygiene to avoid further deterioration of a DFU condition, leads to a more significant financial burden on the patient [20,21,22]. Therefore, intelligent automated telemedicine systems are now the most cost-effective alternative for remote DFU detection. Current DFU detection works have proposed using computer vision methods based on fundamental image processing approaches, supervised classical machine learning, and deep learning algorithms [23,24,25,26,27]. To segment wounds, these methods depend on the detection of edges and morphological processes and techniques for clustering that use distinct color spaces. In terms of medical imaging and computer vision, there are three popular tasks that are addressed by researchers: classification, localization, and segmentation, as shown in Figure 3.

The recent works involving DFU classification, localization and segmentation have encountered major challenges [28,29,30]. These challenges include the costly and time-consuming processes of dataset collection and its expert labeling; the differences in the visual appearances of DFUs due to the lighting conditions in the images and the ethnicities of the patients; high inter-class similarities between DFU skin, healthy skin, and various other skin lesions; and intra-class dissimilarities based on the classifications of DFUs. Therefore, with all the major therapeutic challenges involved in DFU detection, it is the need of the hour to study state-of-the-art works and assess their quality and effectiveness [31,32,33]. Thus, before devising an algorithm and implementing it for common practice, it was best to review the pre-existing approaches. This paper discusses some of the most recent works associated with DFU identification, emphasizing their advantages and limitations.

### Detailed Comparative Analysis of the Related Works

**Figure 4:**
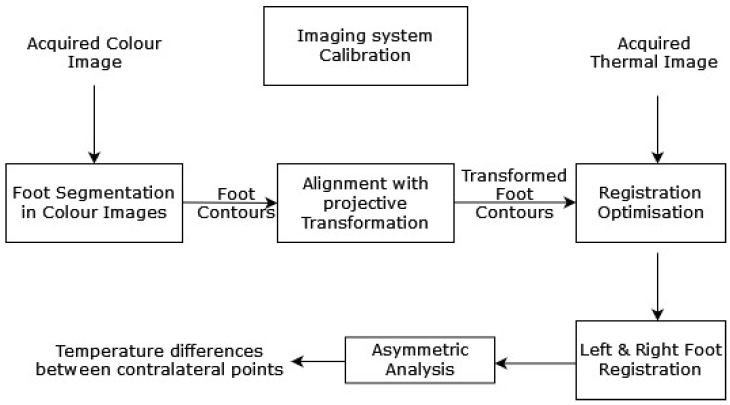
The above-discussed approach for DFU detection [34].

Liu et al. (2015) [34] proposed an automatic diabetic foot complication detection mechanism with an infrared and RGB camera setup. A capturing device was used to detect the presence of DFU conditions and acquire thermal images. An asymmetric analysis, with segmentation and non-rigid landmarks between the registration of both of individual’s feet, was conducted to detect DFUs [35]. The asymmetric analysis was performed in three steps: first, a segmentation approach was used to extract both feet from their background. Then, registration of both feet was completed with the corresponding areas. Finally, the temperatures of the associated areas were compared to determine if the difference was larger than a threshold. A systematic model diagram of the proposed work by Liu et al. is shown in Figure 4.

The proposed approach used K-means and expectation-maximization segmentation techniques. It achieved 97.8% sensitivity and 98.4% specificity scores. Although the proposed approach achieved significant results, one of its primary limitations was that if one foot had already been amputated, the approach could not detect a DFU on the other foot. Further, detection was normally missed if both feet had similar complications.

Dataset: The foot images were captured using a two-stage setup combining a thermal camera and an RGB camera. The patients were from Hospital Group Twente Almelo, Netherlands. A total of 76 images of feet of images with the standard setup.

**Figure 5:**
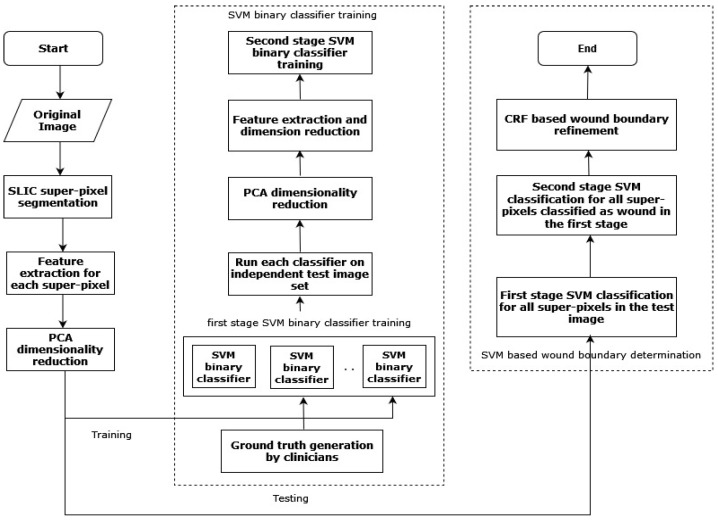
The flowchart of the above-discussed approach [36].

Wang et al. (2016) [36] proposed a two-stage cascaded SVM classifier-based technique for determining the wound area in a DFU. After super-pixel segmentation, the classifier was used in two phases with the SLIC algorithm for color extraction and texture descriptors from the DFU images. In the first phase, K binary SVM classifiers [37] were used on different subsets of training images to find wrongly classified samples. The second SVM classifier ran over the incorrectly classified samples by taking as inputs the color and texture descriptors extracted previously. Figure 5 depicts the flowchart of the approach proposed by Wang et al. [36].

The proposed approach was applied to a smartphone platform and the achieved sensitivity score was 73.3%. However, the proposed system depended on various image-capturing conditions such as illumination, placement of the foot, and capturing distance.

Dataset: The images were collected from 15 patients at the UMASS medical school in the United States over two years. There were 100 images of feet captured by an image capture box that maintained standard illuminations. The images were down-sampled to 560 × 320 for use in the smartphone platform after a Regions of Interest (ROIs) extraction.

**Figure 6:**
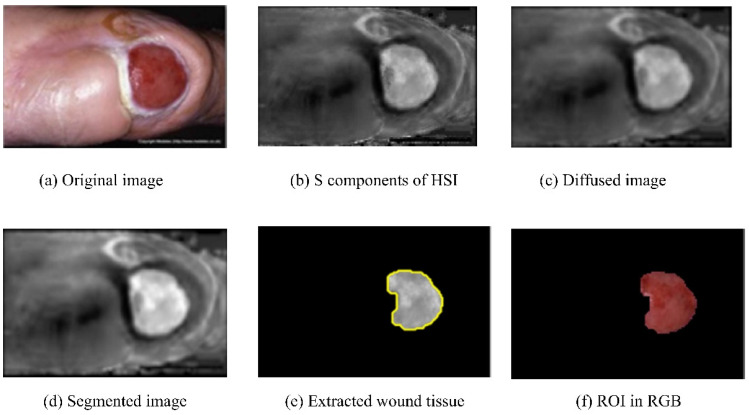
Results of the different components of the above approach [38].

Patel et al. (2017) [38] proposed a step-by-step architecture to detect DFUs. The architecture consisted of pre-processing the images and segmentation and feature extraction, followed by texture detection and classification of the processed images. The input RGB images were converted into HSI color spaces in preprocessing, and the noise was removed using diffusion. Next, image segmentation was performed using multiple algorithms, such as differential evolution, edge detection with a Gabor filter, and region growing, to separate the ROIs from the background. The results of the initial segmentation and ROIs extraction are shown in Figure 6. Next, the texture and color features were extracted, and finally, classification was completed with the extracted features using the K-means algorithm [39]. The K-means algorithm categorized the input images into three clusters: granulation, slough, and necrotic tissue. However, the work did not include the dataset description. The classification results invalidation in the evaluation metrics was another work limitation.

**Figure 7:**
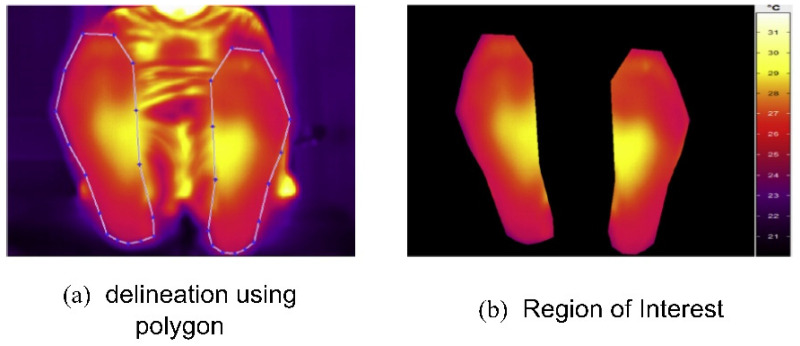
Segmentation results of plantar foot images [40].

**Figure 8:**
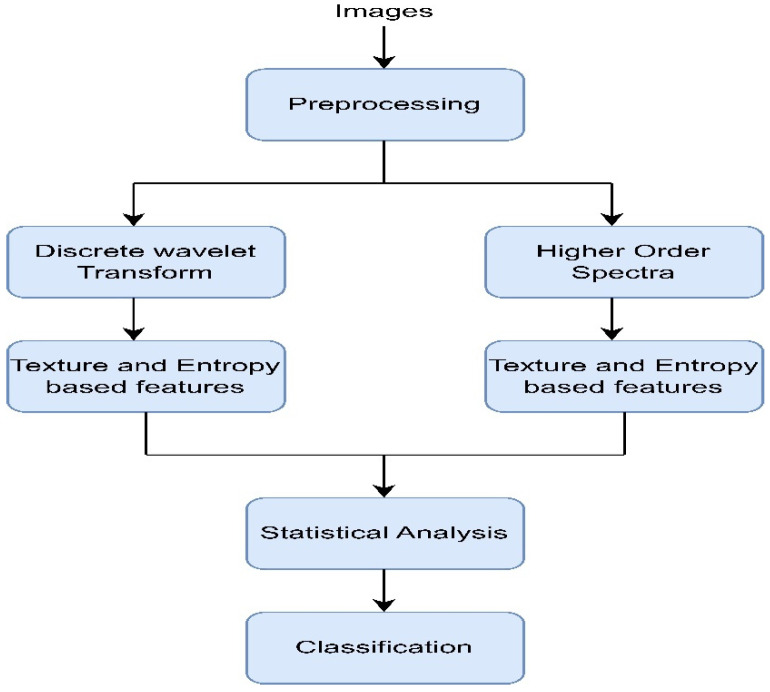
Flowchart of the above-discussed approach [40].

Adam et al. (2018) [40] used plantar foot thermograms from the Diabetes and Metabolism Centre (DMC), Ngee Ann Polytechnic, and Singapore General Hospital (SGH) and designed a computer-aided system to identify the DFUs. At first, the foot images were decomposed with the help of higher order spectra (HOS) and discrete wavelet transform (DWT). Next, several features (e.g., GLCM, Hu moments, LBP, LTE, and entropies) were extracted and fed to the radial basis function kernel (the SVM classifier) to perform classification [41]. The intermediate results after segmentation are shown in Figure 7. The proposed method, shown in Figure 8, attained an accuracy rating of 89.39% while maintaining a good sensitivity score. However, using 33 samples from each normal and abnormal class made the system less reliable for use in real-life scenarios.

Dataset: The thermograms were collected from two separate sources. There were 33 healthy subjects’ images obtained from Ngee Ann Polytechnic and Singapore General Hospital (SGH). The same number (33) of non-neuropathic diabetic patients’ foot images were collected from the Diabetes and Metabolism Centre (DMC) under standard conditions. There were 15 females in both the normal and abnormal groups, and there 18 males in each group that were considered for this study. The average age was 51.94 ± 11.25 years for the normal population, whereas the average age for the diabetes group was 56.18 ± 14.71.

**Figure 9:**
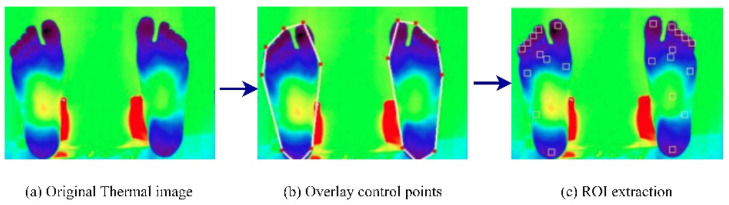
Regions of interest extraction process [42].

Vardasca et al. (2018) [42] proposed an approach that used infrared thermal images and a KNN classifier to perform DFU identification. The images were acquired from the Centro Hospital do Porto, EPE, in standard temperature and humidity conditions. At first, the ROIs were extracted from the input thermal images. The results of the ROI extraction are shown in Figure 9. Then, the KNN classifier, with a K-value of five, achieved an accuracy rating of 90.8% when the KNN was implemented from scratch [43]. However, the authors concluded that using data from only 56 patients made the system less reliable for practical use in DFU prevention. In addition, the lower score in predicting positive classes (28%) required further exploration.

Dataset: The dataset comprised 56 infrared thermal images collected from the Centro Hospital do Porto, EPE. The samples were collected at room temperature (approximately 25 °C) and <50% humidity. An infrared camera (FLIR A325sc with an FPA sensor of 320 × 240) was used to capture the samples.

**Figure 10:**
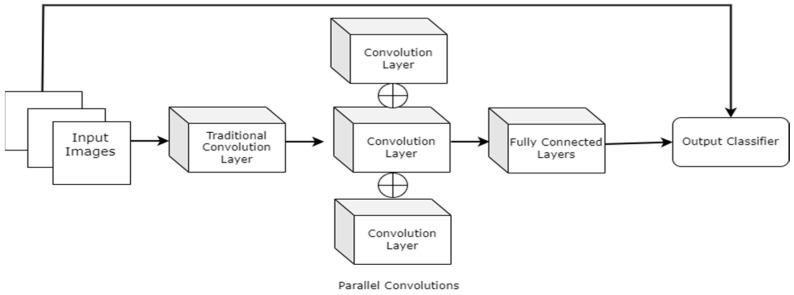
A higher level view of the DFUNet architecture [44].

Goyel et al. (2018) [44] created a new DFU-related dataset for normal vs. abnormal binary classification. The investigations were carried out to understand the characteristics of normal and abnormal skin patches from a computer vision perspective. Several current ML and DL techniques have extracted features from healthy and DFU skin patches. The researchers also proposed a novel CNN architecture (DFUNet), shown in Figure 10, to extract features and perform classification. The proposed architecture consisted of 5 × 5, 3 × 3, and 1 × 1 kernels with parallel connections, and the first traditional convolution layers had kernel sizes of 7 × 7. A detailed experimental study suggested that the proposed DFUNet outperformed the traditional ML-based feature extraction methods and standard CNNs architectures such as LeNet [45], AlexNet [46], and GoogLeNet [47] in normal vs. abnormal classification. The proposed DFUNet achieved an accuracy rating, F1 score, and sensitivity rating of 0.925 (±0.029), 0.939 (±0.024), and 0.934 (±0.033), respectively.

Dataset: The samples were collected from the Lachainchar Teaching Hospital (LTH), United Kingdom, and they consisted of 397 full-foot images (292 abnormal and 105 normal). They were obtained with the help of image-capturing devices such as a Nikon D3300. The images were captured in a parallel orientation, maintaining a 30–40 cm distance from the wound area. As a result, the medical experts delineated the Regions of Interest (ROIs), and 1679 skin patches (641 and 1038 abnormal) were produced.

**Figure 11:**
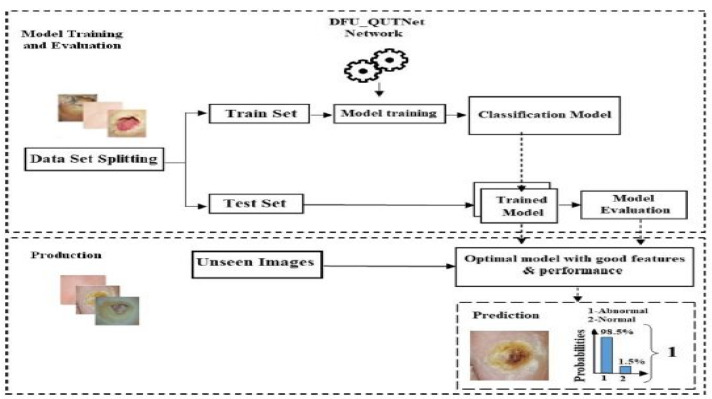
Pipeline of the DFU_QUTNet DFU prediction system [48].

Alzubaidi et al. (2018) [48] introduced a new DFU dataset and proposed a novel CNN architecture for classification. The proposed CNN architecture was named DFU_QUTNet. The proposed architecture’s primary aim was to increase the network width while maintaining a lower depth. KNN and SVM classifiers performed the classifications using the features retrieved by the proposed DFU_QUTNet. Figure 11 depicts the proposed system’s working pipeline.

Further, the proposed model’s results were compared with those of standard CNN architectures (i.e., GoogleNet, VGG16 [49], and AlexNet). The SVM-based classification with features extracted by the DFU_QUTNet architecture obtained the highest average F1 score of the tested architectures (94.5%). Although the proposed model was proven to be significant in this problem domain, it will be interesting to see how it performs with a fully connected network at the end.

**Figure 12:**
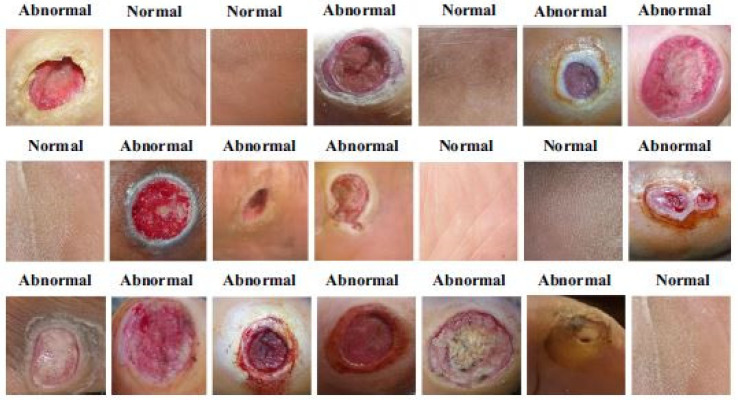
Example images of the dataset [48].

The dataset consisted of healthy and abnormal DFU images acquired from Nasiriyah Hospital’s diabetic center in Iraq. The images were captured using mobile devices in standard conditions, and the images were preprocessed to make them homogeneous. After expert labeling and ROIs extraction, the final dataset consisted of 1609 skin patches (542 normal and 1067 abnormal). Samples from the dataset are depicted in Figure 12.

**Figure 13:**
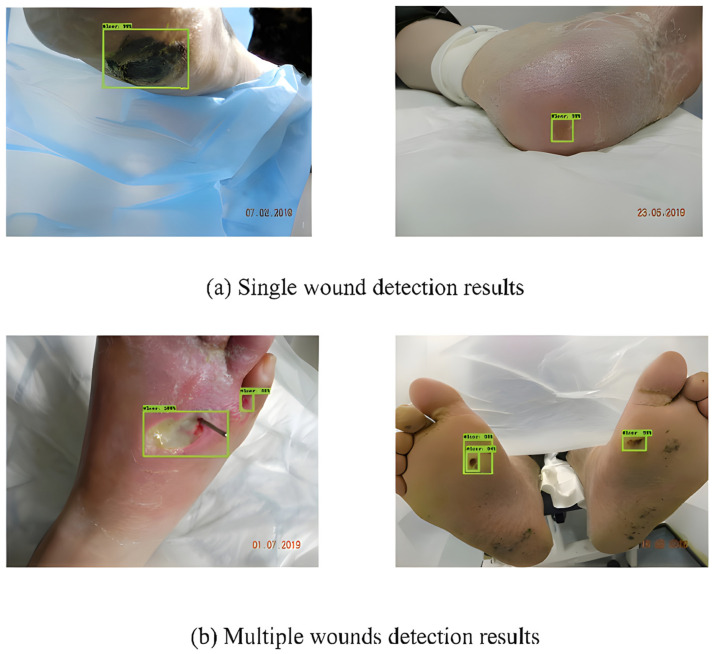
Detection results using EfficientDet [50].

To detect DFUs, Bill Cassidy et al. [50] used Faster-RCNN [51], Inception-v2-Resnet101, FRCNN Inception-v2-ResNet101, YOLOv5, and EfficientDet. They discovered that these networks were significant in terms of producing promising findings. Furthermore, the analysis of the results suggested that among all the considered networks, EfficientDet achieved the highest mAP score (0.6929). Some of the detection results by EfficientDet are shown in Figure 13.

Dataset: The DFUC2020 dataset consisted of 4000 natural RGB images (2000 for training and 2000 for testing). An additional 200 images were provided as the validation set. The samples were gathered over a period of several years by Lancashire Teaching Hospitals (LTH) in the UK. The digital cameras captured the samples at 30–40 cm from the object. The initial samples were heterogeneous. Therefore, the samples are resized to 640 ± 480.

**Figure 14:**
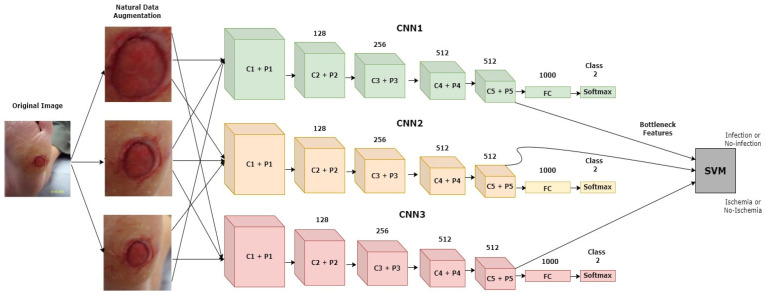
Diagrammatic representation of the discussed model [52].

Goyel et al. (2020) [52] introduced a new dataset with ground truth labels for ischemia and infection recognition. They used multiple traditional ML-based feature extraction techniques and CNN architectures to recognize ischemia and infection as two individual binary classification problems. The diagrammatic representation of the proposed approach is depicted in Figure 14. The ensemble of the CNN-based approach achieved the highest-scoring results for both tasks. However, for infection vs. non-infection, the results were not as promising compared to the ischemia vs. non-ischemia classification results. The highest average accuracy rating and AUC score for infection recognition achieved by the ensemble CNN approach were 73.70% and 73.1%, respectively. Using segmentation subtasks was a great help for achieving better classification results.

**Figure 15:**
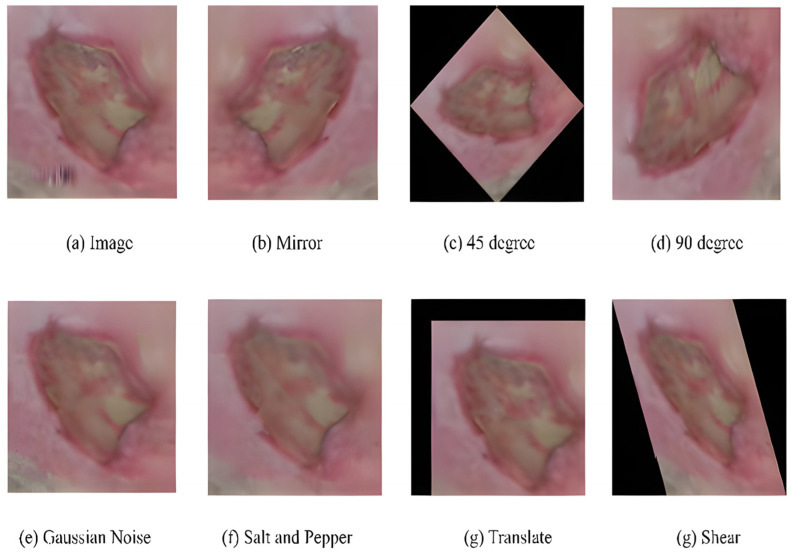
Examples of natural augmented images for a sample [52].

Dataset: The dataset contained two sub-directories of natural RGB images obtained from LTH, UK: one for recognizing ischemia and the other for recognizing infection. Initially, the ischemia dataset included 1459 full-foot pictures (210 with ischemia and 1249 without). Then, 1666 patches were extracted while keeping the ROIs in mind. Finally, natural augmentation was performed, and the dataset builder created a sum of 9870 augmented image patches with a uniformly distributed classes of 4935 (ischemia (1)/non-ischemia (0)). Similarly, the initial number of full-foot pictures in the infection dataset was 1459 (628 infections and 831 non-infections) and 1666 patches were created. Figure 15 depicts examples of the augmented images. The final infection dataset included 4890 augmented patches, with 2945 images included individually for the infection (class ‘1’) and non-infection (class ‘0’) classes.

**Figure 16:**
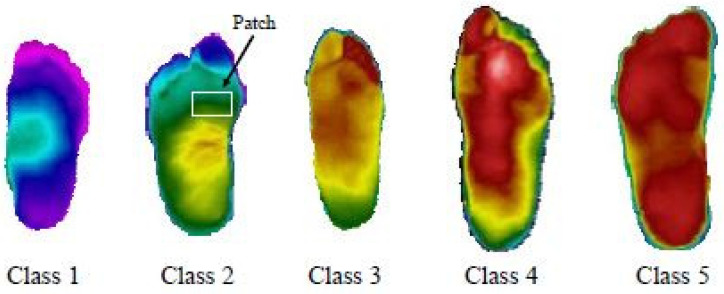
Differing grade levels for DFUs [53].

Cruz-Vega et al. (2020) [53] proposed a model to perform multiclass DFU classification using thermograms. There were five different classes, as shown in Figure 16. The experiments were conducted in multiple setups. Results were achieved using traditional SVM and ANN classifiers and pre-trained GoogLeNet and AlexNet. However, the classification results could have been more satisfactory.

Therefore, a new CNN architecture was proposed, and it consisted of multiple convolution layers with kernel sizes of 7 × 7 and 3 × 3. The proposed shallow network, DFTNet, had significantly improved DFU classification, with an average F1 score of 0.9457. The layer-wise architecture of the proposed DFTNet is shown in Table 1. However, using fuzzy entropy measures and differential evolution optimization in segmentation slowed the model down. In addition, the use of fewer thermogram (110 samples) examples demands further exploration for designing such an approach.

Dataset: The dataset consisted of 167 plantar thermograms, out of which 122 were collected from diabetic patients and the rest (45) were collected from a non-diabetic population. The samples were acquired over a period of three years from multiple medical facilities, hospitals, and institutions in Puebla, Mexico.

**Figure 17:**
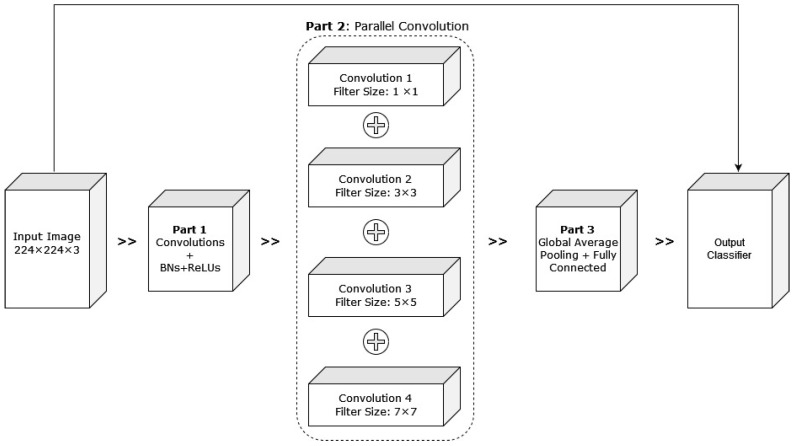
Pictorial representation of the above-discussed approach with four branches [54].

Alzubaidi et al. (2021) [54] proposed hybrid CNNs combining traditional and multi-branch parallel convolutional layers. Multiple versions of the proposed network have been evaluated with different depths in a similar setting. In addition, the convolutional layers in parallel connection had different-sized kernels, which enabled better feature extraction. The general structure of the proposed approach with four branches is shown in Figure 17. The proposed hybrid CNN architecture achieved 95.8% average F1 scores. Although the proposed hybrid CNN architecture achieved promising results, it will be interesting to see how fine-tuning the network parameters can contribute to improving its performance.

Dataset: The dataset contained 754 foot images from Nasiriyah Hospital’s diabetic center in Iraq. The samples were rescaled to 224 × 224 for experimental purposes. The images were split into two categories: healthy or normal skin vs. unhealthy or abnormal skin.

In the MICCAI DFUC2021 challenge [55], a multiclass classification problem was introduced with image samples from the following classes: none, infection, ischemia, and both. The best scores were obtained by BiT-ResNeXt50 and EfficientNet-B3, which were trained on multiple data folds. The highest average AUC achieved by the proposed model was 88.55%. However, its low scores in other evaluation metrics, such as F1 score (62.16%) and recall (65.22%), demands further exploration and the design of more sophisticated CNNs to perform multiclass classification in DFU research.

Dataset: The DFUC2021 dataset consisted of natural image (RGB) samples from four categories: 1703 images of infections, 152 images of ischemia, 372 samples with both ischemia and infection conditions, and 1703 controlled images. The dataset also consisted of 1337 unlabeled DFU foot skin images. The samples were collected from LTH, UK in standard conditions. Two medical professionals completed the ground truth labeling of the images. Additionally, data augmentation was performed to enhance the number of samples in the dataset. The final volume of the dataset became 15,683 (11,689 labeled and 3994 unlabeled) DFU patches.

The comparisons of the proposed approaches for DFU identification will provide a clearer picture to readers. Comparisons could be made in terms of the proposed methodologies. We divided the approaches into conventional machine learning-based and advanced deep learning approaches. We observed, as shown in Table 2, that until 2018, traditional approaches for DFU identification were more popular. However, in 2018, many advanced deep-learning approaches were proposed. In most traditional ML approaches, specific image modalities are used, such as thermal images. These approaches are similar in that they extract image features and use them in ML classifiers to make decisions. Multiple image processing features, such as GLCM, Hu moments, LBP, LTE, and entropies, are used to acquire knowledge from image data. At the same time, segmentation techniques have proven to be very helpful to traditional ML approaches for conducting DFU identification. However, in advanced DL approaches, the requirements of expensive image acquisition techniques are reduced. The evolution of DL learning techniques, especially CNNs, to handle natural image classification has proven to be significant. In most approaches where CNNs are used to identify DFUs, authors have used standard CNN architectures or their own proposed CNNs. However, one interesting point is that standard CNNs, such as Residual Network, Inception Network, etc., have inspired the proposed CNN approaches.

Therefore, further research scope has been created for the exploration of the design and development of a more sophisticated CNN approach to improving these results. However, in medical imaging, various attempts have been made to blend handmade and CNN-based features [50,56,57,58,59] in other illness detection. These visual qualities aid in detecting color and texture cues when utilizing various CV, ML, and DL algorithms for automatic DFU assessment. A comparison of the above-discussed approaches is provided in Table 2.

## 2. Conclusions and Research Direction

The objective of the article was to provide readers with a clear idea of the current work status of automatic DFU identification. The advances in ML and DL approaches have been proved to be a great help to clinicians for decision-making. The application of engineering solutions in DFU identification are relatively new compared to other similar problem domains. Therefore, the most important published works since 2015 are discussed in detail. Observations have been made that the traditional ML and advanced DL approaches are used to solve the problem. However, advanced DL approaches, especially CNNs, have proven to be significant for achieving promising results. Therefore, the major findings from work were: firstly, it introduced the problem to readers so that they can realize the necessity of involving advanced engineering solutions. Secondly, until now, detailed work has been performed to address the challenges and required tools and materials for approaching the problems in DFU identification. We have included details about the approaches and materials. Thirdly, discussions about the advantages of each existing approach and its limitations are reported. This will help readers to find directions for solving such problems.

Further, a comprehensive study of the methods used for DFU detection and the performance metrics displayed by these frameworks in recent works provided evidence for its future scope and challenges. Because of the constant upsurge in the number of diabetes patients and, consequently, the number of cases of DFUs, the need for expert podiatrists and health professionals is rising exponentially. Furthermore, the costly and lengthy procedures for DFU detection and treatment makes it even more challenging to control the cases of DFU. Hence, there is a need to develop an automated system based on computer vision techniques to create a cost-effective, reliable, and user-friendly healthcare solution for identifying diabetic foot ulcers. Nevertheless, the currently available works by researchers performed quite well in identifying DFU skin, though certain loopholes exist in most of these frameworks.

The frameworks discussed in this paper form the future targets that can be addressed by researchers as follows:

(a) An automatic annotator is introduced to make the expert labeling process less challenging.
(b) The performance of these architectures must be further improved to increase their reliability. Once complete, they can be implemented for the detection of other skin lesions.
(c) Implementation of these architectures on mobile devices such as smartphones should be made easier and with improved inference speed, which will increasing their scalability.
(d) Diabetic foot care systems that can operate remotely outside hospitals should be established. In addition, diabetic foot monitoring systems could be introduced in home settings to keep track of a patient’s exposure to the risk of amputation.
(e) These deep learning architectures can be used to analyze and classify the different tissues within an ulcer bed to further develop precautionary recommendations for detecting early key pathogenic abnormalities in diabetic feet.
(f) The development of DL-based pre-processing techniques for the separation of image features such as skin color, wrinkles, moles, and skin deformities from the actual DFU features will allow for better segmentation.
(g) Researchers can conduct error analyses on the widely varied DFU image datasets for different lighting conditions to studying the effects of light on DFU detection by these architectures and minimize errors. They can also analyze and compare the impacts of the capturing devices and make detection more robust.

These state-of-the-art architectures can transform outdated clinical approaches to DFU diagnosis into highly advanced, remote, cost-effective, and user-friendly telemedicine software that can be used globally for detecting DFUs in diabetic patients. This can save significant time while removing the dangers of inaccurate diagnosis and delayed treatment. In addition, the increased workloads of expert medical practitioners due to the shortage of diabetic foot experts and podiatrists can be mitigated with such an automated solution. Further, designing automatic DFU identification approaches for other subtasks such as analysis and identifying biomarkers and how they contribute to DFU identification can be related research domains that researchers can work [60,61].

## Figures and Tables

**Table 1:** The architecture of DFTNet [53].

Layer No.	Layer Type	Filter Size	Stride	No. of Filters	FC Units
1	Conv.	7 × 7	1 × 1	32	
2	Max-Pool	3 × 3	2 × 2	-	
3	Conv.	1 × 1	1 × 1	64	
4	Conv.	3 × 3	1 × 1	64	
5	Max-Pool	3 × 3	2 × 2	-	
6	Conv.	3 × 3	1 × 1	32	
7	Max-Pool	2 × 2	2 × 2	-	
8	Conv.	3 × 3	1 × 1	32	
9	FC (Fully conn)	-	-	-	Class Nos.

**Table 2:** Summary table of DFU identification.

Year	Approach	Advantages	Research Scopes
2015 [34]	Asymmetric analysis of feet from thermal images with the help of K-means and expectation-maximization segmentation approach	The proposed approach could achieve promising sensitivity and specificity in diabetic foot complication detection	Costly setup and experience required to operate; in addition, it missed identification on alternate feet if one foot had already been amputated or if both feet had similar complications
2016 [36]	Cascaded two-stage SVM classifiers with SLIC based a super-pixel segmentation approach	The approach was applied to smartphone platforms to detect DFU wound areas in an offline mode	The detection accuracy depended on the image-capturing conditions such that the illumination, capturing distance, and foot position had to be consistent
2017 [38]	The use of preprocessing, multiple segmentation algorithms, texture and color feature extraction, and K-means clustering	Such a classification approach to categorize the DFU images into different severity classes can provide great help to involved clinicians, helping to build a better treatment strategy for patients	The details of the dataset used were not included in the published work; in addition, the classification results invalidation in terms of the evaluation metrics was another limitation of the work
2018 [40]	Multiple image processing features (GLCM, Hu moments, LBP and LTE, and entropies) with an SVM classifier	The use of multiple feature descriptors helped in looking at the input images in various scales	Although the proposed approach achieved promising results, the use of 66 samples made the system less reliable for real-life scenarios
2018 [42]	Multiple thermal parameters such as the mean, median, and standard deviation temperatures of the ROIs with KNN classifiers	Analysis with multiple K-values provided the proposed system with significant importance	The problem could not be considered as a binary classification; therefore, the use of other classifiers such as SVM or ANN was not possible
2018 [44]	A new DFU dataset was created and a new CNN architecture (DFUNet) was proposed with parallel convolution layers	Multiple ML-based features were extracted, and a detailed results analysis was provided for the standard CNNs (e.g., LeNet, AlexNet, GoogLeNet)	The proposed DFUNet could be trained on various optimizer settings; additional intermediate transition layers in the DFUNet could improve the classification results
2019 [48]	A novel CNN architecture (DFU_QUTNet) was proposed to extract the bottleneck features, and the extracted features were fed to KNN and SVM classifiers to perform classification	The proposed width CNN architecture could extract abstract features by maintaining less network depth; as a result, the network’s training time became shorter	The SVM classifier outperformed the KNN; a completely linked network at the end of the DFU_QUTNet, on the other hand, may improve the results
2020 [50]	Inception-v2-Resnet101, FRCNN Inception-v2-ResNet101, YOLOv5, and EfficientDet were used to detect DFUs	The analysis of results from various standard architectures created a new benchmark result in DFU detection	A new CNN architecture was proposed to perform DFU identification
2020 [52]	Introduced new datasets for ischemia and infection identification; an ensemble of standard CNN architectures (Inception-V3, ResNet50, and InceptionResNetV2) with an SVM classifier was proposed	The proposed super-pixel color descriptor for feature extraction and the feature ensemble of multiple standard CNN architectures helped with extracting significant feature abstraction	The proposed ensemble approach performed well for ischemia identification; however, the approach performed poorly at a comparatively harder infection identification task
2020 [53]	A shallow CNN architecture (DFTNet) with multiple convolution layers with kernel sizes of 7 ×7 and 3 ×3	The proposed architecture was very simple and shallow but outperformed the standard CNN pre-trained models GoogLeNet and AlexNet	Fuzzy entropy measures and differential evolution optimization in segmentation made the model slow
2021 [54]	A hybrid CNN combining traditional and multi-branch parallel convolutional layers	The convolutional layers in parallel connection had different-sized kernels, which helped with better feature extraction	Parameter fine-tuning of the proposed hybrid CNN could be completed to improve the results
2021 [55]	In the DFUC2021 challenge, multiple approaches were introduced to set up benchmark results on the multiclass classification problem of DFUs; the predefined classes were ischemia, infection, both, and none	The best scores were obtained by BiT-ResNeXt50 (Big Image Transfer) and EfficientNet-B3, which were trained on multiple data folds	The benchmark results set by the DFUC2021 challenge demands further exploration of DL-based architecture to improve classification performance

## Data Availability

The data availability statements are available in “Diabetic Foot Ulcers Data”, which can be found at http://www2.docm.mmu.ac.uk/STAFF/M.Yap/dataset.php, accessed on 4 February 2023.
